# A Review on Technologies for Localisation and Navigation in Autonomous Railway Maintenance Systems

**DOI:** 10.3390/s22114185

**Published:** 2022-05-31

**Authors:** Masoumeh Rahimi, Haochen Liu, Isidro Durazo Cardenas, Andrew Starr, Amanda Hall, Robert Anderson

**Affiliations:** 1 School of Aerospace, Transport and Manufacturing, Cranfield University, Bedford MK43 0AL, UK; masoumeh.rahimi@cranfield.ac.uk (M.R.); i.s.durazocardenas@cranfield.ac.uk (I.D.C.); a.starr@cranfield.ac.uk (A.S.); 2 Network Rail, Milton Keynes MK9 1EN, UK; amanda.hall@networkrail.co.uk (A.H.); robert.anderson4@networkrail.co.uk (R.A.)

**Keywords:** localisation, sensor fusion, railway maintenance, autonomous systems

## Abstract

Smart maintenance is essential to achieving a safe and reliable railway, but traditional maintenance deployment is costly and heavily human-involved. Ineffective job execution or failure in preventive maintenance can lead to railway service disruption and unsafe operations. The deployment of robotic and autonomous systems was proposed to conduct these maintenance tasks with higher accuracy and reliability. In order for these systems to be capable of detecting rail flaws along millions of mileages they must register their location with higher accuracy. A prerequisite of an autonomous vehicle is its possessing a high degree of accuracy in terms of its positional awareness. This paper first reviews the importance and demands of preventive maintenance in railway networks and the related techniques. Furthermore, this paper investigates the strategies, techniques, architecture, and references used by different systems to resolve the location along the railway network. Additionally, this paper discusses the advantages and applicability of on-board-based and infrastructure-based sensing, respectively. Finally, this paper analyses the uncertainties which contribute to a vehicle’s position error and influence on positioning accuracy and reliability with corresponding technique solutions. This study therefore provides an overall direction for the development of further autonomous track-based system designs and methods to deal with the challenges faced in the railway network.

## 1. Introduction

**Figure 1:**
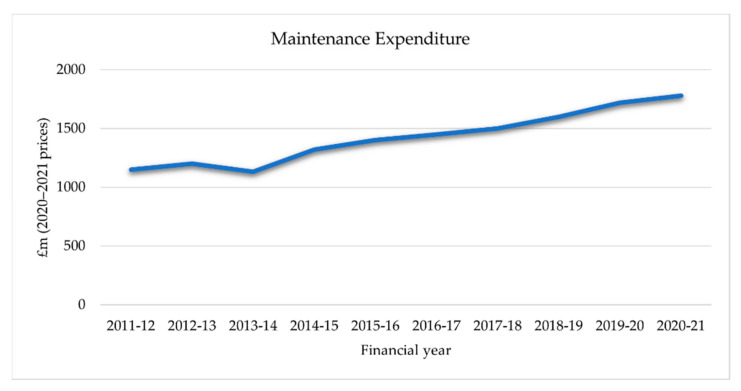
Total maintenance expenditure, 2011–2012 to 2020–2021 (2020–2021 prices).

Rail transportation demand is steadily increasing over the world, particularly in metropolitan regions with rapidly growing populations. Even in Europe, where population growth is slower, estimates show an increase in the proportion of people who travel by train. This causes national railways infrastructure maintenance and renewals costs across a total of around 300,000 km of combined track and exceeding €25 billion per annum across Europe [1,2]. Harnessing data and analytics could help European rail infrastructure operators to better target their maintenance spending more productively. In Great Britain’s rail network, there are 40,000 bridges and tunnels, 9000 level crossings, and 9941 miles (16,000 km) of railway tracks. It has been reported [3,4] that in Great Britain the rail network experiences 4.7 million train journeys every single day, demonstrating the importance of railway infrastructure. Railways require regular maintenance to ensure safe operating conditions. It costs over £1 billion per annum in the UK, accounting for 18% of Network Rail’s overall expenditure [5,6]. The high-level quantitative information relating to maintenance is depicted in Figure 1, which shows the trends in total maintenance in terms of 2020–2021 prices. Maintenance expenditure has been steadily increasing since 2013–2014 and is expected to reach over 1830 million pounds in 2020–2021.

Therefore, preserving or improving the safety, reliability, and quality of the whole railway system is a key challenge, and is paramount for passengers, employees, and the entire rail network. Without reliable rail track maintenance, the safety of the rail network will be at risk, and delays would occur regularly. For these reasons, innovative maintenance solutions for railway systems, as well as the integration of maintenance into operations, are constantly studied and developed to ensure a better management of the railway network. 

Today, different industry sectors benefit from automation, which has led to the development of a number of robotic solutions to maintain and repair applications in the industry. For instance, miniature robot models have been applied in different maintenance tasks, including in highways, aircraft servicing, underwater facilities, power line maintenance [7], fault detection [8], and track cleaning or performing repair jobs such as 3D printing [9,10]. A wheeled robot with a manipulator can undertake a variety of dangerous and remote operational tasks such as tunnel inspection [11] or the cleaning of nuclear reactors [12]. They could also be utilised in the inspection and maintenance of railway tracks [13]. Automation and computational intelligence techniques can dramatically improve the efficiency and effectiveness of maintenance. This guideline additionally applies to the railway maintenance industry [14]. 

Many different robots have been designed to do various railway track maintenance tasks; however, most of them are limited to specific scenarios, uses, or applications [15]. To perform the intended tasks, autonomous robots, as with all other technical systems, must meet certain requirements that vary depending on the individual application or task. 

Autonomous systems will be one element involved in solving the trade-off between the transport capacity challenge and the maintenance cost and time reduction. Rail infrastructure managers from all over the world are interested in developing automatic inspection systems that can detect rail flaws, sleepers’ irregularities, and missing fastening elements as high-speed railway traffic grows. These systems can improve the ability to detect defects and minimise inspection time, allowing for more frequent railway network maintenance. The condition of the railway track is also monitored as part of the maintenance strategy [16]. Currently, industries use equipment for inspection and maintenance activities separately; these two activities have not yet been merged. The introduction of artificial intelligence (AI) and cognitive analytics technologies into these two procedures can make the entire process dynamic and autonomous [15]. 

In this paper, firstly, the role of robotic and autonomous systems in track maintenance are discussed as well as the objectives and the related maintenance techniques. Following that, a number of challenges related to the autonomous maintenance vehicles are reviewed and various ways of positioning on the railway track are investigated. Next, sensors and their functions in localisation as well as several fusion approaches aimed at enhancing positioning accuracy on the railway track are pointed out. Finally, the sources of uncertainty contributing to errors in railway location systems are highlighted.

## 2. Railway Maintenance Objectives and the Related Techniques

### 2.1. Maintenance Policy

Rail track maintenance encompasses all technical and administrative actions aimed at inspecting, repairing, and maintaining railway tracks in order to keep trains moving smoothly and securely while also extending their service life [17,18]. The following are the primary objectives of railway track maintenance:

1. Due to the high speed of trains, heavy axle loads, and repetitive loads, the track structure’s strength continues to deteriorate.
2. The track structure is subjected to various degrading factors such as rain, sunlight, and wind. The deterioration of rolling stock and rails is unavoidable.
3. The track structure has to withstand so many other curvatures, speeds, and load effects, particularly at curves, points, and crossings.

For these reasons, it is critical to maintain railway track on a regular basis. Railway maintenance tasks, however, are costly, and poor maintenance or an inability to conduct preventive maintenance will result in severe consequences. As a result, the application of robotic and autonomous systems in this area is proposed to undertake these maintenance tasks with higher accuracy and reliability. According to the conducted research in [19], the majority of robotic and autonomous systems advances in railway inspection and maintenance areas are related to the rolling stock and rail track, with 56% and 28%, respectively. The cost-effectiveness of robotics automation in railway track maintenance and related tasks have been already proven [20]. 

Today, track maintenance—including replacement, track stabilization, ballast injection (stone blowing), sleeper replacement, tamping the ballast, excavation, spiking rail, tightening bolts, and aligning the track—is achieved through the utilisation of highly specialised machines [21]. A number of these robots are massive and can conduct more than one task, for instance, ballast tamping combined with track lining and leveling. However, in order to get the best result for each part, it is decided that a specific robotic system should be applied for each task. As a result, each individual robot will concentrate on a single target for doing a highly specialised and efficient job. 

In terms of railway track maintenance, a fully autonomous robotic system detects and removes sleeper bolts, feeds new fastener, and assembles them in high-speed train lines [22]. Rowshandel et al. [23,24] suggested an integrated robotic system comprising of a mechanised trolley, a robot, a commercially available alternating current field measurement (ACFM) device, and a laser distance sensor for the highlight precise identification and characterisation of surface-breaking defects in rails. Auto-Scan [25] is another example of an autonomous rail inspection system. It is an autonomous trolley that detects defects on the railway track using electromagnetic acoustic transducers (EMATs). RailPod [26], a commercially available autonomous rail inspection robotic platform, can run both on and off track, but only on plain surfaces, because it has both rail and pneumatic wheel reconfigurable mechanisms. 

A robust rail condition monitoring methodology was proposed in [27], in which a laser scanner mounted on a moving rail vehicle to detect track fractures, scorings, and excessive wear was applied. An autonomous track geometry diagnostician’s computer system which flags poor track locations was proposed in [28]. Missing or broken track components including bolts, clips, ties, tie plates, anchors, and turnout components have also been detected using robotic and autonomous systems [29]. For instance, a new method for detecting missing or defective rail fastener problems has been proposed in previous research [30] which uses the histogram of oriented gradients information and a mixture of linear Support Vector Machine (LSVM) classifiers. 

Besides providing infrastructures with an autonomous maintenance vehicle, proper maintenance of railway assets is also necessary and should be done periodically. This can be periodic updating, periodic comprehensive maintenance, regular inspection, and key repairing of track [31]. Services are at significant risk of failure if they are not properly maintained, with negative consequences for user satisfaction and asset professionals’ performance. As a result, two types of maintenance techniques have been established by National Rail to keep the tracks as functional as possible: preventive and corrective railway maintenance. Both demand a significant amount of resources, specialised equipment, and well-trained personnel [32].

#### 2.1.1. Preventive Maintenance

**Figure 2:**
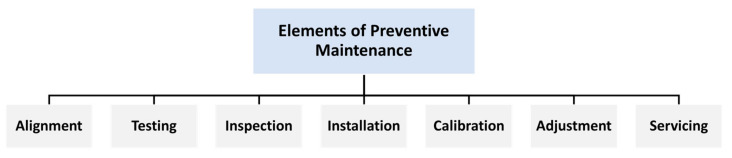
Elements of preventive maintenance.

Preventive maintenance (PM) is an important part of maintenance activity. An integral aspect of PM is systematic inspection, detection, and repair of incipient failures, either before their occurrence or before they proceed to a failure state, by competent persons involved in maintenance [33,34]. The main goals of PM are to extend the useful life of capital equipment, reduce critical equipment breakdowns, improve the planning and scheduling of needed maintenance works, reduce production losses due to equipment failures, promote the health and safety of maintenance personnel, and provide maximum system reliability and safety with the least amount of maintenance resources [35]. PM is divided into the following options, as shown in Figure 2:

1. Inspection: comparing physical, electrical, mechanical, and other properties (as appropriate) to the expected standards to evaluate the serviceability of materials/items.
2. Servicing: cleaning, lubricating, charging, preserving, and so on, of items/materials on a regular basis to avoid incipient breakdowns.
3. Calibration: determining the value of an item’s attributes on a regular basis by comparing it to a recognised standard with known accuracy.
4. Testing: testing or checking out on a regular basis to verify serviceability and discover electrical/mechanical degradation.
5. Alignment: changing the stated variable aspects of an item in order to achieve optimum performance.
6. Adjustment: periodically modifying specified material variable parts in order to achieve optimal system performance.
7. Installation: regular replacement of limited-life parts or equipment experiencing time cycle or wear degradation to maintain the stated system tolerance.

Preventive maintenance for these operations takes place at specified intervals or according to certain criteria to limit the likelihood of equipment failure or degradation in their functioning. Because of the structure of a rail track, each element must be considered, as mispositioning of one of them might result in poor alignment and, in the worst-case scenario, train derailment. As a result, it can be divided into two parts: day-to-day maintenance (scheduled continuously) and seasonal maintenance [36].

- Day-to-day maintenance:

The goal of this type of maintenance, from first inspection to final control, is to get the track operational and running as soon as possible. Three main units are mostly used for maintaining the track: stoneblowers, tampers, and Dynamic Track Stabilisation (DTS) machines [37]. These machines are employed for large-scale refurbishment projects, while a vast fleet of other units operates for smaller tasks. Stoneblowers and tamping machines act on the ballast and the rail. These three devices allow the track to be repositioned and reinforced. The purpose of the operations is to fix the track’s position by shifting the ballast and adjusting rails and sleepers. However, maintenance also entails the removal of defects, particularly from the rail. As previously stated, the rail is prone to many failures as a result of the train’s passing and the force exerted, and if not addressed promptly, the defect might spread and lead to the disintegration of the rail [38]. The most common rail problems are head check, gauge corners, cracks, and squats, which begin on the rail surface but spread deeper if trains continue to run [38]. This can be kept in check by routinely grinding the rail surface and lubricating the joint between the rail and the wheels.

- Seasonal Track Maintenance:

Weather is another factor that that has a significant impact on the train’s operating conditions. Temperature changes, leaves, plants, and ice can either damage the track or limit the train’s abilities. The functional operation of the track, as well as the safety of passengers, are significantly influenced in this circumstance [39]. Various operations are carried out to address this problem depending on the weather conditions. In the winter, for instance, rails are prone to freezing and ice formation; in this situation, scraping or blowing hot air and then spraying hot liquid on the rail will prevent the rail from freezing [38]. In autumn, leaves on the rail reduce the friction between the wheels and the rail, causing friction to increase and the train’s wheels to hardly adhere to the rail, especially during acceleration phases. In this situation, the Rail Head Treatment Trains (RHTT) blowing high pressured air would remove the leaves. It then adds an adhesion gel to the wheels to strengthen their grip on the rail. Workers are also meant to cut down the trees that are suspected of colliding with the train. Plants on the railway track begin to grow in the summer, causing the track’s structure to become unsettled. In this case, herbicides are sprayed on the track to kill the seeds, and vegetation is pruned back to maintain the accessibility of the track. Another issue in the summer season is temperature. As rails are thermal conductors, they retain the heat. As a consequence, they expand and are no longer in their proper position [38], meaning that trains are subject to derailment. To prevent this failure, some parts of the rail are painted in white to reflect the sun’s rays. However, in other circumstances, preventing expansion is insufficient, and the rail tension is adjusted to allow the track to dilate and stretch without buckling.

#### 2.1.2. Corrective Maintenance

Corrective maintenance (CM) is the task of locating, isolating, and resolving a fault so that failed equipment can be substituted or restored to an operational condition within the tolerances or limits set for in-service operations [33]. It plays a significant role in the efficiency of maintenance organisations. Sometimes, the track is too damaged to be restored or upkeep may be too costly [32]. In this case, corrective maintenance—which consists in replacing the degraded pieces [34]—is undertaken. Concerning railway maintenance, two types of operations are carried out depending on the damage level. If only a small portion of rail requires repair—for instance, replacement of a small part—manual operations are done, including cutting out the defective rail part, removing the rail part from the site using the road-rail vehicle, clearing the space around it, bringing the new rail, proceeding to illuminate thermic welding, proceeding to profile and grinding, and inspecting the final rail [32]. However, for more advanced maintenance, specifically designed machines conduct the tasks.

**Figure 3:**
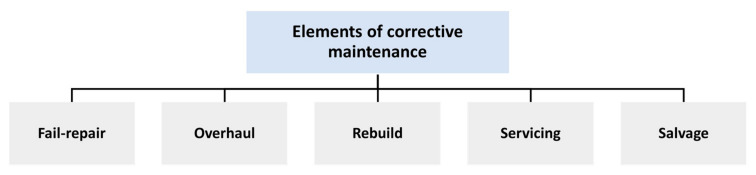
Elements of corrective maintenance.

The CM process takes time to be achieved and requires the use of multiple machines as well as the participation of several personnel. The activities for larger maintenance are almost entirely automated. Track renewal is done when the rails have sustained too much damage. From the dismantling of the previous rails through to the inspection of the new track, a special purpose machine is employed to assure the complete replacement of the track [40]. In total, CM may be classified into five major categories: fail-repair, salvage, rebuild, overhaul, and servicing, as shown in Figure 3:

Overall, maintenance is critical in the railway industry since it ensures both safety and profitability. In the railway industry, there has been a tendency toward increased automation and productivity. As a result, it has become larger, more expensive, and more technically complicated than ever. The track infrastructure is a significant part of the railway system, and its upkeep is a key financial consideration in technical, administrative, and managerial decisions.

Now, in order to do autonomous maintenance, it is necessary to first identify the location of the railway vehicle precisely. Therefore, in the next section, possible approaches are investigated.

## 3. Railway Vehicle Localisation Strategies

Railway vehicle positioning plays a prominent role in the safety of the system since the positioning information is used for train separation and control. Rail vehicles are confined to travel along the railway and an error in train location might result in a dangerous overestimation of braking distance. Therefore, an accurate and reliable estimation of the location of rail vehicles such as trains, trams, subways, and rail-robots is critical to rail-system management [41,42]. A wide range of sensors and infrastructures have been proposed and implemented for railway vehicle positioning, and so their classification is required. These sensors are mostly divided into two classes:

(1) Elements in the railway environment (infrastructure-based)
(2) On-board sensors (infrastructure-less)

### 3.1. Track Infrastructures-Based Strategy

**Figure 4:**
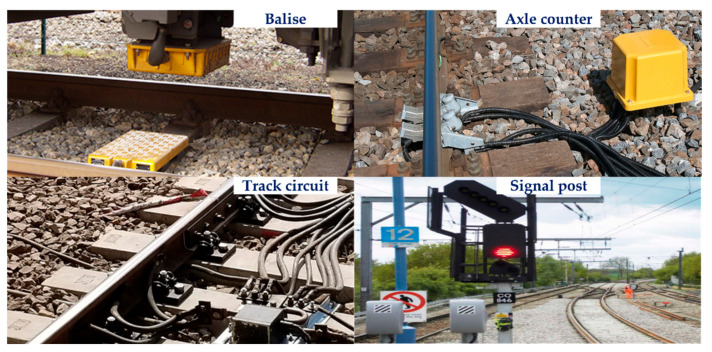
Track-side infrastructures.

Infrastructures in the railway environment are used as a way of determining the location of trains on the railway track. Examples of track-side sensors are magnetic coils, cable loops, contacts, track-circuits, axle counters, transponders, and radio balises [43,44]. A number of these are shown in Figure 4. The presence of a train on rail tracks, for example, is detected using a track circuit (a simple electrical device). This equipment is not devoted to locating the train specifically but rather to locating it indirectly on a track portion. The location can also be determined using detectors installed along the railway, which are used for train protection. These sensors could be transponders (balises), which communicate with the train’s on-board equipment when the train passes over them [45]. At best, this technology can provide train position information with a precision of a few hundred metres, which is sufficient for providing a train safety system, especially when there is a risk of collision. The collision avoidance operations are accomplished by the protection systems with interlocking [41], specifying whether or not a train can access a track section/block. As a result, any future obstruction on the railway track is avoided. However, the main drawback of these localisation solutions is installation and the high cost of maintenance. Because of this, these infrastructures are usually spread along the railway track, with distances ranging from tens of metres for current Radio-Frequency Identification (RFID)-based systems to tens of kilometres for conventional commercial transnational railway systems [41]. In terms of application, balises or cable loops are mostly used in continuous train control systems such as the European Train Control Systems (ETCS) Level 2 or the German LZB (Linienzugbeeinflussung) with the goal of better localising the train [46]. Currently, localisation strategies—for example, those used in subway applications—rely heavily on ground sensor infrastructure such as balises/tags and beacons [47,48]. 

### 3.2. On-Board Sensors

**Figure 5:**
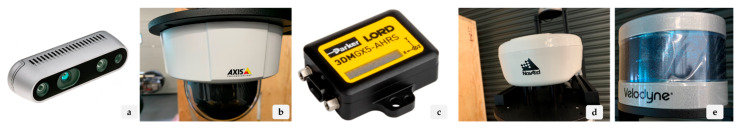
On-board sensors: (**a**) stereo depth camera; (**b**) environmental camera; (**c**) IMU; (**d**) RTK-GPS; (**e**) 3D Lidar.

On-board sensors include tachometers, inertial sensors, satellite-based positioning systems, and other on-board sensors [49]. Several on-board sensors are shown in Figure 5. In terms of train positioning based on these sensors, all the components and computations parts are on-board, and in contrast to infrastructure-based localisation methods, it also requires a map of the railway tracks [50]. The track map contains the tracks’ information and connections, and the measurable features of the track, providing a reliable position estimation. There are multiple approaches to train localisation based on on-board sensors and a map, and they are different in terms of sensor types or combinations, processing methods, and evaluation scopes [50]. A combination of a Global Positioning System (GPS), Doppler measurements, and a track map is studied in [51]. 

Much research has been done in the field of train positioning methods based on GPS. For instance, European Train Control systems (ETCS) level-3 use GPS especially for train integrity confirmation [52,53]. Due to the train’s complex environment, using only GPS cannot afford reliable position data in some specific scenarios such as tunnels, hilly regions, and urban canyons [54]. Therefore, multi-sensor fusion positioning methods have been proposed in this regard [55,56]. A fusion of GPS and Inertial Navigation System (INS) is widely used as a localisation system [57]. This system works properly when there is a continuous correction by GPS; otherwise, there is a cumulative position error caused by INS. In other words, if there is no GPS correction, using the Inertial Measurement Unit (IMU) alone for a long time will result in a huge offset. Therefore, more sensors and information sources are necessary to precisely and consistently locate the train, such as odometers, eddy-current sensors, Doppler velocity sensors and accelerometers, digital maps and wayside transponders such as RFID or balises, and other on-board equipment [58]. The necessity of augmenting GPS with other measurements for operational and safety reasons is discussed in [59,60].

A multi-sensor scheme that collects data from various sensors installed on the vehicle (specifically, an IMU and a GPS) and performs a Kalman-based filtering recursion is investigated in [61]. That study focused on solutions that could be used on every rail vehicle regardless of the ground equipment on the specific lines. In another study [62], an adaptive multi-sensor data fusion technique for the precise assessment of the train’s position and velocity, based on three on-board sensors—longitudinal accelerometer, odometer, and GPS receiver unit—is suggested. A localisation technique for railway vehicles, based on the fusion of tachometer and IMU and with the aim of performance enhancement in terms of speed and position estimation accuracy, is introduced in [63]. This fusion was thanks to Kalman filtering (KF) theory. Another piece of research [64] presents a particle filter-based localisation approach for a rail-guided robot. A particle filter was considered to integrate odometry with inertial measurements, laser scans, and image data. As a result, a rail map, a motion model, and a perception model were developed to implement a 1D estimation. 

Dead-reckoning systems such as eddy current sensors [65,66], Doppler radar [67], Inertial Measurement Unit (IMU), and optical imaging [68] are applied for vehicle positioning based on estimating distance and direction of travel from a known fixed point. However, these systems lead to uncertainty due to inherent cumulative errors which are typically caused by wheel slippage, wear of wheels and mechanical parts, bias, and hysteresis, etc. [69]. Therefore, these sensors cannot be utilised alone for long periods of time in safety-critical applications such as collision avoidance and train automation [70], and need to be reset periodically to improve localisation accuracy. For this reason, whether they are used alone or in combination, they must be merged with GPS [71] or track-side markings such as RFID-type devices or balises [67]. The fusion of GNSS, inertial sensors, and odometry was examined in [72]. 

It is worth mentioning that current train-borne localisation systems using GNSS, odometers, and track maps have severe shortcomings concerning accuracy and reliability. The problem is that they cannot always determine immediately which of several parallel tracks the train is situated on. This is the most important prerequisite for the safety of railway vehicle control systems [73]. Therefore, lidar sensors are the most promising choice to complement those systems proposed in train localisation [74]. They are used for identifying large structures and environmental changes (e.g., exiting a tunnel or entering a station) based on a topological map of those features. It can be said that, in the case of train positioning, lidar is related to the topological landmark detection, as in the work of [43]. This information is then utilised to verify other sensors’ location estimates and reset dead-reckoning errors. 

**Figure 6:**
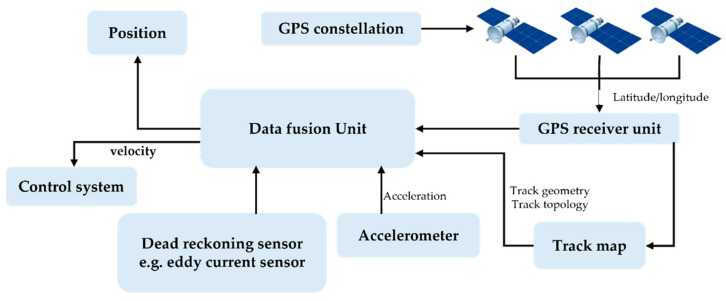
Main components of a railway vehicle positioning.

On-board train localisation from the perspective of safety assessment was investigated in [44]. The researchers attempted to answer the question of how an on-board train localisation system can be designed so that it ensures safe operations. They propose a generic approach that is not reliant on specific sensing devices. The system is based on three sources of information: a GNSS receiver, a velocity sensor, and a digital track map. Figure 6 represents the components of an on-board localisation system that fuses the incoming information from the on-board sensors. It determines the position of railway vehicle without relying on any trackside aids.

In another classification, Durazo Cardenas et al. [54] cited the positioning sensors based on the principles of the sensor’s technology. They gathered four types of general location systems: GNSS [75], radiolocation [76], proximity [77,78] and dead-reckoning systems. A summary of these sensors can be found in [54]. 

In Table 1, a number of on-board sensors and track-side infrastructure are compared with each other in terms of absolute and relative positioning, rate of frequency, long-term and short-term baseline, outage difficulties, and environmental impact. In this table, absolute localisation refers to a global localisation solution which relies on the GNSS constellations or landmarks to restore the position and orientation information with regard to a global reference frame [79]. Relative localisation refers to a local localisation technique which uses on-board sensors and kinematic models to estimate the robot’s pose relative to its initial pose [79]. Baseline is defined by the distance between the rover and a reference point [80]. For instance, in terms of applying GPS, baseline is about the vehicle and the base station. The length of the baseline varies between short, medium, and large [80]. Another parameter in this table is outage issue, which refers to the unavailability of a sensor for a period of time, which can be due to different reasons. For example, GPS could have an outage issue inside the tunnels, near the buildings, or in urban canyons, and so on [81]. The last parameter is environment impact, which includes any changes in the environment such as rain, snow, fog, or any natural change in the environment.

In the following section, firstly, the application of the most used on-board sensors and track-side equipments in a railway system is provided. Next, the main functions, as well as their usual rate of frequency, and their advantages and disadvantages, are investigated.

## 4. Sensor Hardware

As has been previously mentioned, one of the most important challenging tasks within any autonomous driving framework is localisation from data collected in real time. Therefore, accurate and reliable measurement of on-board systems plays a critical role for the vehicles moving on the railway track. There are various sensors for measuring the position and speed of railway vehicles. The most common ways include: tachometers, transponders, balises, INS, Doppler effect, and GPS [69]. A comparative survey highlighted benefits and drawbacks associated with different sensor types [82,83]. These sensors can be evaluated and classified according to several parameters, including cost, accuracy, reliability, sensitivity, coverage, speed of response, and availability [69]. Accurate and reliable odometry information may be achieved using a number of these sensors, whose redundant or complementary data are combined intelligently to produce more accurate and reliable information. In the following, each sensor is described briefly.

- Tachometers:

Wheel angular speed sensors are widely diffused in railway applications due to their resilience and reliability. They are frequently employed as a principal form of speed measurement equipment. Through this type of sensor, when pure rolling conditions occur, the train speed can be simply estimated [84]. Various types of tachometers have been developed and applied, including optical, capacitive, active, and passive electromagnetic tachometers; however, the incremental optical tachometer is more accurate and efficient than the other types. A tachometer’s accuracy is usually impacted by a variety of sources of errors, including noises in mechanical and electrical parts, mechanical imperfections, wheel slip and slide, sampling frequency, and alteration in the wheel diameter as a result of wear and turning [69,84]. A number of approaches for correcting the position inaccuracy caused by slip and slide have been proposed and carried out, including correction by marginal distance, mutual correction of numerous axles, and frequent resetting of the position using transponders [85].

- Transponders:

Passive and active transponders are track-based items that are used in conjunction with on-board odometers (integrating tachometers), representing a reliable method for measuring the position of a train [69]. They have been used by numerous railway operators. The transponders transmit a signal to the train-based receiver that includes information on their position and in some circumstances signaling information. Increasing the number of transponders along the track to reduce the positioning error results in higher costs and lower reliability in the system [69].

- Balise:

A balise is an electronic beacon or transponder that is installed between railway tracks as part of an automated train protection (ATP) system. It provides the train a position reference as well as direction information. It is an example of a transponder fixed on the track to correct the position uncertainty that builds up within the train location subsystem over the time. Balises, as magnetically coupled transponders, do not require a steady energy source [86], and can be termed as passive. The balises are placed at approximately regular intervals, with the distance between them being determined based on two factors: the speed of the trains and complexity of the railway [87]. As it is important for the trains to stop precisely at stations, point zones, and buffer stops, up to eight balises are required at a minimum interval defined by the design [87]. In order to increase the possibility of balise detection by the train and decrease the possibility of the train failing to read a balise, the use of more than one balise in critical stopping locations is considered.

- Doppler Radar:

Based on the principle of the Doppler frequency shift effect, Doppler radar can calculate the train’s immediate speed by analysing the frequency difference between the radar transmitted and reflected wave [88]. It is a non-contact sensor with two microwave antennas that gives accurate and consistent results independent of reflecting surface or vibration [89]. Compared to a tachometer, Doppler radar is found to provide more accurate data [90]. Heide et al. [91], by doing a number of experiments, demonstrated that the use of a coded 24 GHz Doppler radar can provide high precision data (within 20 db) for vehicle position and speed measurement. On the other hand, Malvezzi et al. [92], while discussing odometric estimation for a train protection system, stated that Doppler radar output is often affected by noise and systematic errors. Mirabadi et al. [69] also stated this issue and identified sources of errors including very smooth reflective surface and change in radiation angle owing to acceleration and braking action, vibration, and bias error.

- Inertial Navigation Systems (INS):

INSs are navigational systems capable of measuring the acceleration, speed, and position of a moving train along the stable axes [79]. An INS is a system that is basically composed of at least three gyros and three accelerometers to derive a navigation solution. The accelerometer will measure the acceleration of the vehicle by integrating the acceleration signals both speed and position data. On the other side, gyroscopes are used in order to measure an angular rotation of the vehicle [93]. They can be used to obtain accurate information of the trajectory of the train in a horizontal and vertical direction. Unlike tachometers, which depend on wheel rotations, the INS system is self-contained, and this is a major advantage of this system. They do not need a line of sight such as GPS. They can be used in any weather condition and environment, both underground or overground.

- Global Positioning System (GPS):

GPS is a satellite-based radio navigation technology which is the core functionality for any navigation system [79] and provides absolute position information with a known ratio of error. The fundamental advantage of GPS is its long-term stability and its resistance to the accumulation of errors over time. GPS is mainly utilised for more than simple outdoor navigational tasks and it is effective in areas with a clear view of the sky. However, GPS sensors are ineffective in certain areas such as tunnels, forests, underground, and underwater spaces [94]. They also have outages caused by satellite signal blockage, occasional high noise content, multipath effects, low bandwidth, and interference or jamming. Common GPS sensors are utilised for positioning, which has an accuracy of 10 m. This does not provide sufficient accuracy [95] for train localisation.

- Light Detection and Ranging (LiDAR):

The lidar sensor is an optical device which uses laser light pulses to gather information from surfaces in the form of “points” (3D coordinates). Compared with cameras, the lidar sensor operates more reliably at different weather conditions and is less influenced by the lighting or weather conditions due to the infrared laser that provides an adequate illumination. Therefore, even in tunnels or under bridges, appropriate measurements can be obtained [73]. LiDAR sensors are also widely deployed in railway applications for different reasons, including object detection and collision avoidance, and in level crossings for detecting whether there are passenger cars, trucks, or people [96]. In [74], the application of LiDAR in a train-borne localisation system is investigated. It is mentioned that position measurements at turnouts remain ambiguous. The combination of GPS, velocity sensors, and digital track map are not capable of addressing the challenges of unavailability of GPS in some parts of environments, and velocity sensors can fail to specify which branch at a turnout is taken by a train. In [97], a 2D Lidar is deployed in an underground railway environment to specify high-speed train localisation.

- Visual sensor:

There are various publications related to the deployment of vision sensors in railway inspection and maintenance applications such as the detection of missing bolts, railhead wear, and other surface geometry inconsistencies [98]. A vision system consisting of a monocular thermal camera mounted on a train for detecting the rails in imagery as well as for detecting anomalies on the railway is pointed out in [99]. In another piece of research, a prototype system for railway object detection, installed in the cab of a train, is presented [100]. The aforementioned system consists of a single camera that acquires images of the railway scene in front of the train and a near-infrared laser primarily used to add illumination to the area the camera is directed at when the light is insufficient. A summary of the publications describing traditional computer vision approaches are described in [101]. The advantages of cameras over other active sensors are high data density and visual information, enabling the detection of the boundaries of objects and the classification of these objects precisely. 

In Table 2, some of the positioning sensors, including on-board sensors (infrastructure-less) and elements in the railway environment (infra-structure-based) are mentioned, along with their functions, advantages, and disadvantages.

## 5. Sensor Fusion 

### 5.1. Sensor Fusion Techniques

Sensor fusion is an essential aspect of most autonomous systems; therefore, various types of algorithms and methodologies have been widely researched in recent years and are now well-established in the literature. Due to the diversity and variety of fusion algorithms proposed in the literature, getting the current state-of-the-art fusion techniques and algorithms is a demanding task, according to a recent study [108]. Recently, several reviews on the topic of multi-sensor fusion have been published, with some describing the architectural structure and sensor technologies in AV [109,110], and others focusing on processing stages such as sensor calibration, state estimation, object and tracking [111], [112], or detailing techniques for multi-sensor fusion, such as deep learning-based approaches [113,114].

A review study in [113] divided these techniques and algorithms into two categories: classical sensor fusion algorithms and deep learning sensor fusion algorithms. On the one hand, classical sensor fusion algorithms, such as knowledge-based approaches, statistical methods, probabilistic methods, and so on, fuse sensor data using theories of uncertainty from data flaws, such as inaccuracy and uncertainty [115]. Deep learning sensor fusion techniques, on the other hand, entail the creation of numerous multi-layer networks that enable them to process raw data and extract features in order to accomplish difficult and sophisticated tasks such as object detection. Deep learning is a subset of artificial intelligence and machine learning that can be considered as an advancement of neural networks [113]. The quantity of research into deep learning sensor fusion algorithms in autonomous vehicles (AV) has increased noticeably. Convolutional Neural Network (CNN) and Recurrent Neural Network (RNN) algorithms are among the most used in autonomous vehicle perception systems. To increase the real-time performance of object detection, [116] suggested advanced weighted-mean You Only Look Once (YOLO) CNN algorithms to merge RGB camera and LiDAR point cloud data. Some other examples of deep learning-based sensor fusion algorithms include ResNet (i.e., Residual Networks), a residual learning framework that facilitates deep networks training [117]; SSD (i.e., Single-Shot Multibox Detector), which discretizes bounding boxes into a set of boxes with different sizes and aspect ratios per feature map location to detect objects with variant sizes [118]; and CenterNet [119], which represents the state-of-the-art monocular camera 3D object detection algorithm. 

Despite the vast quantity of research which has been done in the field of environment perception with deep learning approaches, the application of deep learning to localisation has not received the same level of attention or maturity. As a result, there is a lot of promise for using deep learning algorithms, especially RNN, to improve sequential localisation data. Learning algorithms may in the future provide an end-to-end deep learning localisation and mapping system that avoids feature modelling and data association, reducing errors and uncertainties associated with unmodeled dynamics and imprecise modelling [113].

### 5.2. Sensor Fusion Algorithms for Vehicle-Based Localisation on the Railway Track

As has been mentioned, new approaches in instrumentation, technology, engineering, and, in particular, sensor fusion, have opened up new paths for achieving better reliability and accuracy in measurement. Integration of several sensors for speed and position measurement is the concept which has attracted much interest in industry and research departments [92,120]. Fusing measurements from different independent sensors which have different kinds of input/output attributes and characteristics will extract the best information in terms of accuracy and reliability. Therefore, when multi-sensor data fusion techniques are included in the system, the navigation system becomes more robust. In other words, it can increase the robustness of the system against possible faults of each element it is composed of. This can help railway engineers to achieve higher safety.

The use of multi-sensor data fusion techniques have also gained much popularity and been found to be advantageous when used in intelligent transportation systems [121,122]. Integrating different position and speed sensors will give more information about the system conditions for monitoring and control tasks [69]. The combination of sensors should be chosen in a way which optimizes coverage of multiple aspects, including availability, reliability, speed of response, cost, and accuracy of the system, besides providing a better on-board measurement system. For instance, integrating tachometer and transponder sensors, a type of primitive sensor fusion, will increase the accuracy level by re-initiating the position at some fixed points, but it still does not overcome errors caused by slip and slide between two transponders [123]. A localisation algorithm for increasing the accuracy of the odometric estimation, especially in critical adhesion conditions, based on sensor fusion between tachometers and inertial measurement unit, is suggested in [84]. A data fusion technique is proposed in [62] for a train localisation system consisting of three on-board sensors, namely, longitudinal accelerometer, odometer, and GPS receiver unit. Wang et al. [124] proposed a train positioning method which fuses vison and millimeter-wave radar data. The proposed framework includes the loop closure detection part which eliminates the cumulative error when the train detects a key position, and the radar-based odometry part which can realize the positioning of the train on the whole railway line.

In [66], a hybrid framework for locating trains travelling on track routes based on GNSS and eddy current sensor device, implemented by an Extended Kalman Filter method, is proposed. This positioning system performs a robust localisation even in the case of noise or when a sensor fault occurs. In another piece of research [71], two different fusion approaches which use two different system models to approximate the kinematics of train-borne location system are investigated. These are an eddy current sensor device providing the train velocity and a GNSS delivering the absolute position. The first fusion is based on a polar coordinate system model, whereas the second fusion approach uses two cartesian system models with the ability to switch between these models. Evaluating the fusion approaches shows that in case of GPS failures, the first fusion approach with its system model in polar coordinates can propagate the train kinematics more adequately and thus achieve a better performance than the second fusion approach.

Another common fusion approach is based on the integration of GPS and INSs, which typically results in a drift in the estimation of the vehicle speed and position [63]. To address this problem, a filter is used aimed at decreasing the difference between the data output signals coming from INS and another one, e.g., GPS [95]. Due to GPS signal degradation in some parts of the environment, including indoor environments, dense forests, and near the tall buildings, it is necessary to consider alternative solutions to compensate this outage, for instance a fusion of tachometer and IMU (Inertial Measurement Unit). 

**Figure 7:**
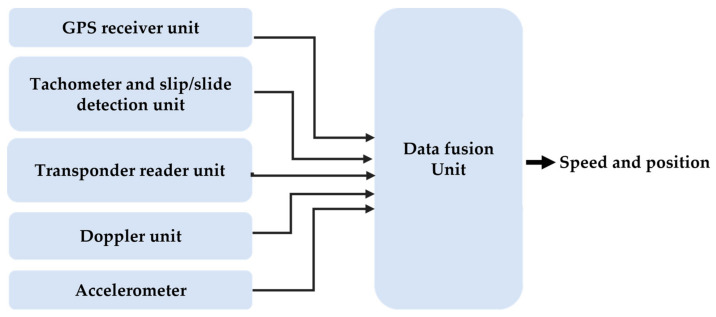
Integration of different sensors for measuring train position and speed.

Figure 7 shows an example of fusion of different sensors for the measurement of train speed and position:

After investigating sensors, their functions, and various fusion approaches applied in railway vehicle positioning, the remaining gap concerns the uncertainty sources which impact the positioning system performance and must be taken into consideration and analysed from different perspectives. In the following section are mentioned in detail. 

## 6. Uncertainty in Railway Localisation Performance

Localising maintenance vehicles, which undertake the inspection and repair of railway track in the global level, leads to specifying the defect positioning in the global level as well. Most of the off-the-shelf localisation systems in railway applications, such as tamping adjustment or track geometry refinement, generally require localisation at meter-level or at several meters, while finding the absolute location (cm level) of railway vehicles would result in specifying the defect location in the absolute level. It would also help the rail-system management, as it can assure the safe running of vehicles on the railway track. In this regard, accuracy and the degree of confidence of the provided position are the main concerns. Therefore, it is of high importance to identify challenges, potential factors, and effects of uncertainties which impact the location accuracy. In the following, five key elements which contribute to a vehicle’s position error, including sensor hardware, environment, information sources, and positioning estimators, are explained [125,126].

- Method performance uncertainty:

Methods used in the position estimation lead to positioning error in several ways. The first item is about validity or integrity of the received information. It is necessary to be sure about the presence of outliers or errors before inputting these data to a positioning algorithm. This is because the calculated positions reflect these wrong data and deteriorate the accuracy of the system [31]. Therefore, input validation should happen as early as possible in the data flow, preferably as soon as the data is received from sensors. 

Moreover, the time-domain consistency, frequency sychronisation between multiple sources, and the timeliness of processing data are essential for the stability of localising.

Another point concerns the strategy considered for data fusion [69]. There are various information fusion techniques, each with their advantages and drawbacks [84]. Therefore, it is important to study the selected fusion method. 

The final point concerns the proper setting of initial conditions and performance parameters for some of the positioning algorithms, especially those employed for data fusion. Setting up proper conditions leads to positioning algorithms working properly. Otherwise, an incorrect selection results in increasing the resulting error instead of reducing it. For instance, in the extended Kalman filter algorithm, proper specification of process-noise-covariance and initial-estimate-covariance can provide an optimal gain, based upon which it assigns a weight to the current state measurement and the prediction state. Bad specification will show a positioning error in the result.

- Sensor hardware uncertainty:

To guarantee a reliable baseline, the hardware systems must be checked physically before being used, because any issues—including receiver failures, calibration failure, or any failure in any of the pieces of the location system, e.g., breakdown of receivers—are considered as sources of error, which can cause severe problems when used in a real system [127]. Sensor calibration is one of the main items which should be checked first, as it minimises any measurement uncertainty and verifies the precision and reproducibility of measurement instruments. Another point concerns the appropriate selection of sensors based on their characteristics and the requirements of the application. For instance, vision sensors such as ASUS stereo cameras are not suitable for outside environments, as they cannot work properly in strong sunshine. The output appears to be noisy from the robot’s perspective as if subject to random error, and the values obtained from the ASUS camera will be unusable [128]. Illumination dependency is only one example of the apparent noise in a vision-based sensor system which need to be taken into consideration before sensor selection. Picture jitter, signal gain, and blurring are all additional sources of noise that potentially reduce the useful content of a color image. In terms of railway applications, sensor specification such as resolution, frequency, and sensing range should be considered as well. For instance, the maxiumum sensing range for a stereo camera is 10 m, and it might fail at detection in the case of a fast-moving vehicle; fast speed causes a blur motion problem in camera imaging.

Another factor is sensor aliasing; even when applying noise-free sensors, the amount of information is generally insufficient to identify the vehicle’s position from a single percept reading. Therefore, techniques must be employed that base the vehicle’s localisation on a series of readings and, thus, sufficient information to recover the robot’s position over time [128].

- Pre-process uncertainty:

The number and quality of the information sources used for position estimation also have a direct impact on the positioning estimation error. It is necessary to provide real-time, reliable, and precise information for train positioning as it has a direct impact on vehicle safety and control, collision avoidance, and autonomous driving systems. One of these sources is the transmission rate of each technology. Based on the strategy followed by the position estimator, ranges of some received signals can be obsolete by the time they are used. Furthermore, time synchronisation of the received information is essential, as it can cause an offset in the detection of the signal and add extra error, especially in time-related positioning algorithms [15]. Sensor fusion would be a challenge in this step. For instance, to address this challenge, a novel fusion approach is proposed in [129]. This method is based on the Kalman filter, which can handle asynchronous data. 

Moreover, the intrinsic accuracy related to each technology makes the position error range from a few meters in the case of using GNSS, to several hundred meters, e.g., Global System for Mobile Communications-Railway (GSM-R). Therefore, based on the nature of the sensors, and their features and functions, their stand-alone or fusion use can be considered.

Position data, generally from GPS, has varying levels of accuracy, and data from different sensors are often not well aligned spatially. This misalignment happens because of GPS position error, such as multipath reception, which is especially problematic when the GPS receiver does not have a clear view of the sky. In this regard, an alignment strategy as a pre-processing step is proposed to mitigate GPS error [129].

- Environment uncertainty:

Environment is another factor that impacts the error related to the location of a system, especially on coverage and accuracy. In the case of railway vehicle localisation, the environment would be a changing factor as the vehicle moves from cities to open areas, where existing infrastructure will be different. Therefore, the environment would result in different error ratios in vehicle location, while information sources and estimation algorithms are the same. The estimated position can be more or less accurate depending on the number and the geo-localisation of the infrastructure in the environment. These infrastructures refer to the number of satellites in line-of-sight with the receiver, as well as track-side objects such as the balises [87].

Based on the features of the terrain or the orography, the coverage and accuracy of the different technologies will be limited. For instance, ingress to tunnels, dense forests, tall buildings, and passage through deep and narrow track openings significantly degrade or even block the reception of the signals temporarily. The best example in this regard is concerns GPS signals, which may not always be available in parts of the environment such as tunnels, hilly regions, canyons, and so on [130]. It might be the case that the signals are available, but that environmental features including satellite signal blockage, occasional high noise content, multipath effects, low bandwidth, and interference or jamming would impact the accuracy of the position estimate.

**Figure 8:**
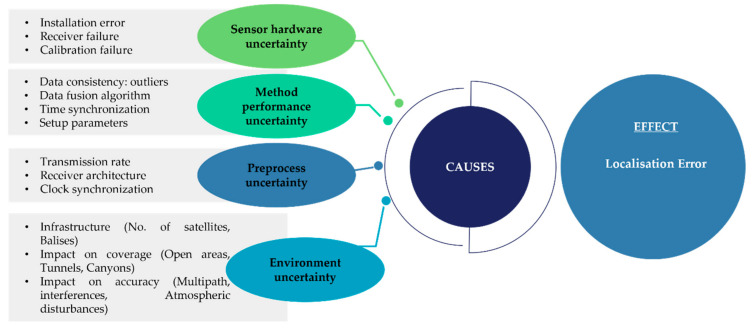
Fishbone diagram showing factors contributing to location errors.

The main causes of inaccuracy and errors that affect the estimation of a position are shown in Figure 8:

## 7. Conclusions

The periodic inspection and maintenance of railway track assets is critical to the safe operation of infrastructure and the management of the continual degradation and ageing of the assets. Autonomous and reliable methodologies can facilitate cost-effective and efficient asset management. Because of the rapid progress made by robotics and autonomous systems in the railway maintenance sector, developing intelligent asset management strategies for digitalisation and smart management for rail infrastructure is a path towards the intelligent industrial 4.0. Precise and real-time rail vehicle localisation is essential to robotic command and control, task execution, safety and efficiency. 

As a result, the current study gives a comprehensive overview of the hardware and software methodologies required for autonomous vehicle positioning on the railway track for maintenance purposes. The following list overviews the main topics which have been covered in this review paper:

- First, the railway infrastructure maintenance requirements and strategies, the maintenance objectives, and the general preventive and corrective workflows are reviewed, revealing that accuracy in localisation is essential for autonomous inspection and repair systems.
- Secondly, a review of the most recent and relevant railway vehicle positioning approaches, based on infrastructures in railway environment and on-board sensors, with their principles, advantages and disadvantages were highlighted. It was identified that applying trackside positioning strategy not only lacks efficiency and accuracy for real-time applications, but also requires large civil investment for construction and successive maintenance.
- Next, for obtaining a comprehensive perception for accurate localisation, the sensor fusion techniques and algorithms were discussed to review the applicability of different sensing methods. The most recent fusion approaches based on machine learning were also discussed. It was also mentioned that deep learning fusion approaches are mostly applied in perception, and that further research in pose and depth estimations, loop closure detection, and feature descriptors is needed to achieve maturity in localisation and mapping.
- Furthermore, the uncertainty sources in railway vehicle positioning were discussed to address the challenge features from different sources which impact the localisation accuracy and reliability. Each uncertainty source was separately investigated and the solutions and strategies to mitigate the impact of that source were also provided.

This review provides a comprehensive discussion of the challenges of localisation technologies for railway maintenance vehicles. It provides an overall reference for localisation system architecture design for both autonomous systems and manual railway rolling stocks.

## Figures and Tables

**Table 1:** Train Positioning Sensor Characteristics.

Type	Positioning Sensors	Usual Rate of Freq.	Absolute Positioning	Relative Positioning	Long-Term Solution (Large Baseline)	Short-Term Solution (Short Baseline)	Outage Issue	Environmental Impact
On-board sensors	IMU [49]	100 Hz	No	Yes	No	No	No	No
	Wheel sensor (tachometer or odometer)	10 Hz	No	Yes	No	Yes	No	Yes
	GNSS [49]	20 Hz	Yes	No	Yes	Yes	Yes	Yes
	Eddy current Sensor [49]	N/A	No	Yes	No	No	No	No
Track-side equipment	RFID	N/A	No	Yes	No	Yes	No	Yes
	Balise	N/A	No	Yes	No	Yes	No	Yes

**Table 2:** Advantages and disadvantages of sensor used in rail applications.

Category	Sensor	Function	Usual Sampling Frequency	Advantages	Disadvantages
On-board sensor (infrastructure-less)	Tachometer [69]	Measuring the rotational speed of a machine.	20 Hz	High short-term accuracy, efficiency, and reliability	Low resolution, electrical noise, impacted by mechanical imperfections such as backlash, polynomial accuracy degradation in the presence of slip and slide between the train wheel and track
	INS [54,69]	Tracking the position and orientation relative to a known starting point	~100 Hz	High short-term accuracy and reliability, not subject to interference outages	Polynomial accuracy degradation, error accumulation over time
	GPS [54]	Suppling an absolute position information in world coordinates	1 Hz	High short-term accuracy and reliability in most outdoor environments, available and relatively inexpensive to implement	Outage in tunnels and performance degradation in urban canyons, affected by poor weather conditions and other sources of interference, dependency on external signal providers
	Wheel encoders [54]	Estimating the position of the vehicle by counting the number of revolutions of the wheels that are in contact with the ground (a relative positioning technique)	~20 Hz	Simple to determine position/orientation, short term accuracy and allows high sampling rates, low-cost solution	Position drift due to wheel slippage, lower sensor resolution, surface irregularities, error accumulation over time, velocity estimation requires numerical differentiation that produces additional noise
	Doppler radar [102]	Calculating the immediate speed of the train	N/A	Overcome the slippage of the vehicle, work reliably at speeds up to 350 km/h, work for speed and distance measurement	Does not work properly in winter on snowy tracks, often affected by noise and systematic errors
	Eddy current sensor [103,104]	Able to detect inhomogeneities in magnetic resistance along the track, e.g., rail clamps or switch components as well as irregularities of the rail	N/A	Provide precise noncontact and slipless speed measurement of rail vehicles, drift-free, unbiased measurements, robust enough to withstand weather influences, dirt, and daytime	Frequency is based on speed, cannot provide real-time high accuracy position
	LiDAR [73]	Emitting laser light pulses to gather information from surfaces in the form of “points”, as well as object detection	~10 Hz	High resolution, large field of view, the ability of providing robust ranging data for object detection and localisation, operating more reliably at different weather and ambient illumination conditions	Reflection of signal wave is dependent on material or orientation of obstacle surface, Expensive solution, affected in extreme weather conditions such as heavy snow, fog, or rain
	Vision sensor [101]	The most accurate way to create a visual representation of the world	~20 Hz	Providing huge information that can be utilised to generate steering control signals for the mobile robots, images store a huge meaningful information, provide high localisation accuracy, inexpensive solution	They influence by varying ambient lightening conditions especially in outdoor environments, and severe weather situations such as fog, snow, and rain, fail to provide the depth information needed to model the 3D environment, requires image-processing and data-extraction techniques, high computational cost to process images
Elements in the railway environment (infrastructure-based)	Balise (an electronic beacon or transponder) [102,105]	Determining the absolute positioning of a rail vehicle along the track, allowing determining the direction of movement	N/A	Do not require contact or direct line-of-sight between the identification tag and the reader device, needs no power source	Compatibility and not universal for every network
	RFID [102,106]	Used for the purpose of tracking and identification of the location of individual rail vehicles or wagons at all times	N/A	High momentary accuracy and reliability at intermittent locations, work effectively where the continuous signaling system is not present	Materials such as metal and liquid can impact signal, sometimes not accurate enough or reliable as barcode scanners, expensive, implementation can be difficult & time consuming
	Track-circuits [78,107]	A safety-critical asset that determines which sections of track are occupied by trains, ensure the safety of rail traffic	N/A	Very simple to maintain	Can delay trains because the signaling system is designed to fail to a safe state, electronic circuits are more vulnerable to lightning strikes, restrictions on placing impedance bonds

## Data Availability

No data are associated with this article.
